# Distal Femoral Varus Osteotomy for the Valgus Knee after Distal Femoral Growth Plate Fractures in Children: A Case Report

**DOI:** 10.1155/2019/2091932

**Published:** 2019-12-22

**Authors:** Ryo Kanto, Hiroshi Nakayama, Shinichi Yoshiya

**Affiliations:** Department of Orthopaedic Surgery, Hyogo College of Medicine, 663-8501 1-1 Mukogawa-cho, Nishinomiya City, Hyogo, Japan

## Abstract

An 18-year-old male suffered a valgus injury to the right knee due to a fall during a bigfoot race he took part in when he was 15 years old. He visited a different hospital at the age of 15. No obvious ligament injury or fracture was noted on MRI and physical examination. However, he gradually became aware of the valgus deformity of the right knee. Finally, he could not take part in a sports activity because of right knee pain. X-ray images at the age of 18 at an initial visit to our department showed severe valgus deformity with mechanical lateral distal femoral angle (mLDFA) of 71 degrees in contrast to left mLDFA which was 87 degrees. We performed a biplane-cut distal femoral varus osteotomy (DFO). Postoperative X-ray images showed an improvement of 86 degrees in mLDFA. Bony fusion was achieved six months after surgery, and he could play several sports activity.

## 1. Introduction

Fractures of the distal femoral growth plate are remarkable in that they are the third most common growth plate fracture in children (after wrist and ankle), yet they carry a risk of growth disturbance in up to 90% of cases [1]. Sports-related injuries and motor vehicle accidents are currently the most common causes of injury. Proper care of these injuries includes counseling patients and parents regarding the future likelihood of growth-related complications. Among them, Salter-Harris (SH) type II is the most common, making up about half of growth plate fractures, whereas types IV and V are rare, accounting for only a few percent [2]. Distal femoral perichondral ring injury (SH type VI, Rang's type VI) is a relatively rare injury and is known to lead to a high prevalence of growth disorders with angular deformity [3]. However, the timing of treatment and approaches to treatment have yet to be established. Here, we report a case of distal femoral osteotomy for a valgus knee after a distal femoral perichondral ring injury (Rang's type VI) in a growing male patient.

## 2. Case

**Figure 1:**
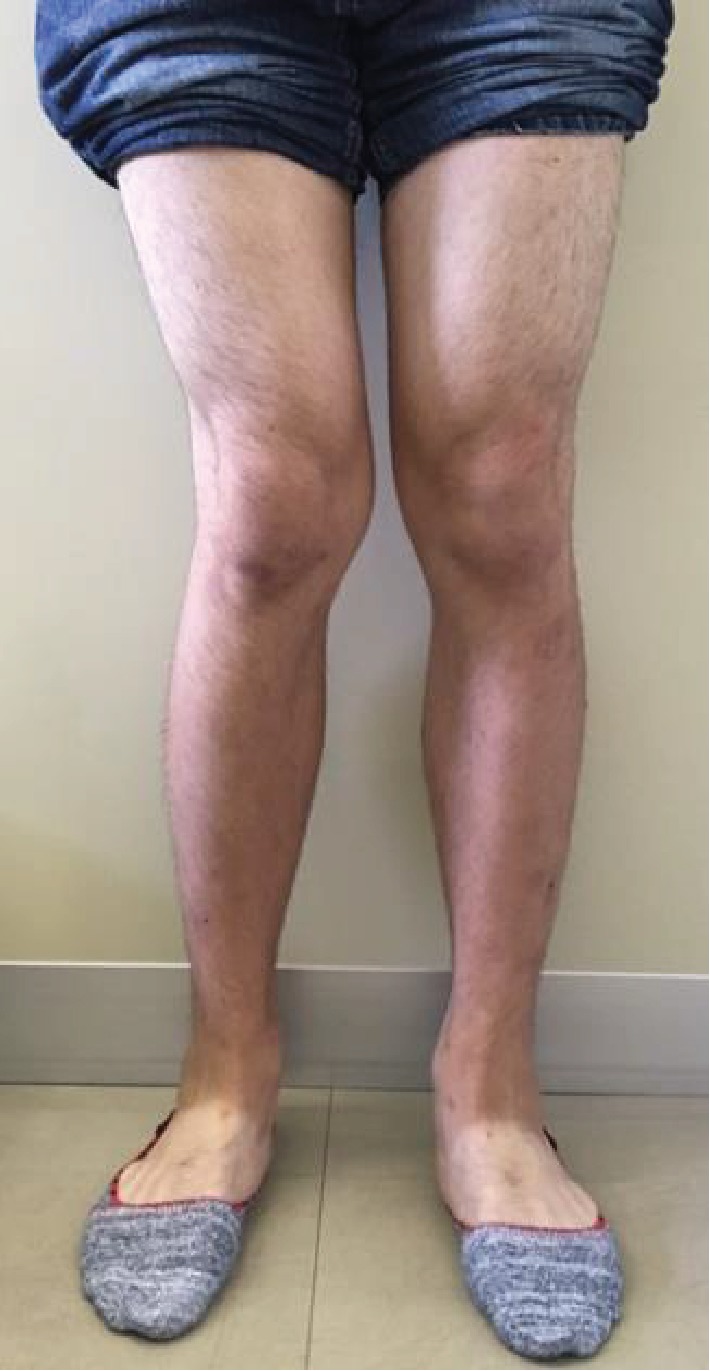
At the time of the initial visit to our department, visible valgus deformity of the right knee is noted.

**Figure 2:**
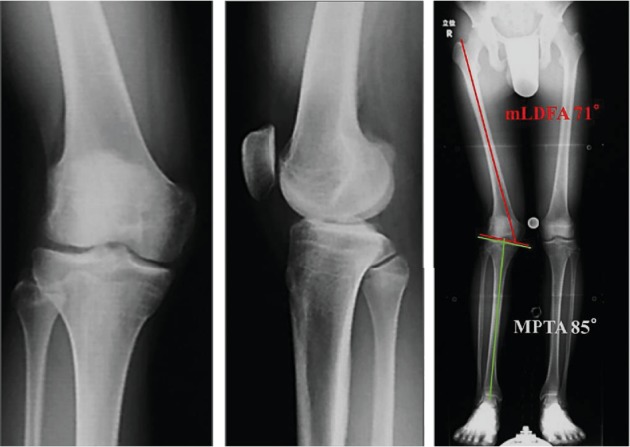
Simple X-ray images taken during the initial visit to our department. Severe valgus deformity is noted with an FTA of 160 degrees and a %MA of 100% from the medial edge of the tibial plateau.

**Figure 3:**
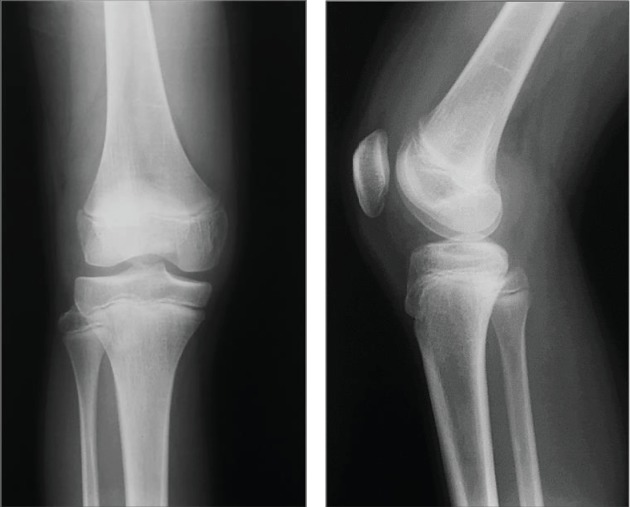
Simple X-ray images at the time of injury (age 15). There are no obvious fractures or growth plate fractures.

**Figure 4:**
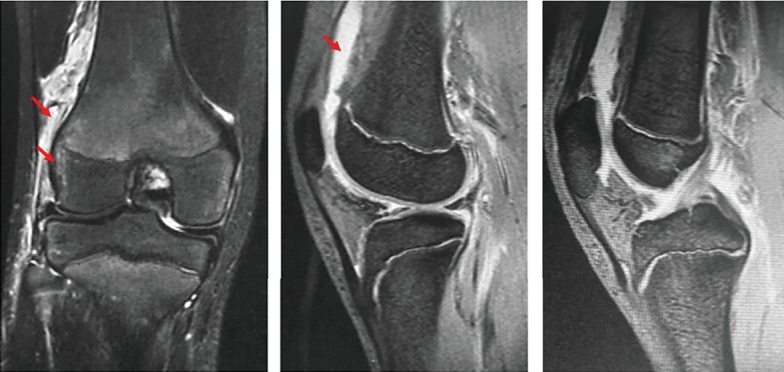
Simple MRI (T2-enhanced) at the time of injury. Mild bone marrow edema in the lateral condyle of the femur and a high signal area suggestive of hemorrhage under the muscular layer are noted.

**Figure 5:**
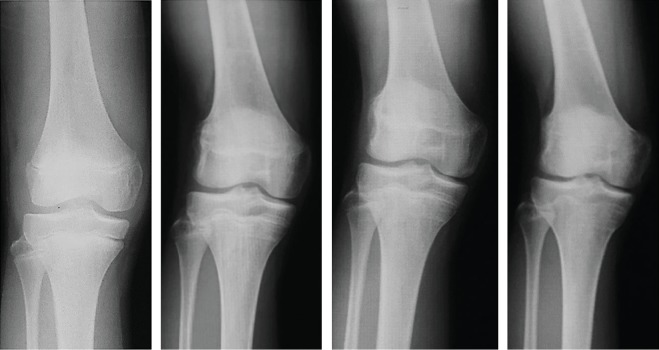
X-ray images taken during the four years after the injury showed that the valgus deformity was gradually progressing each year.

**Figure 6:**
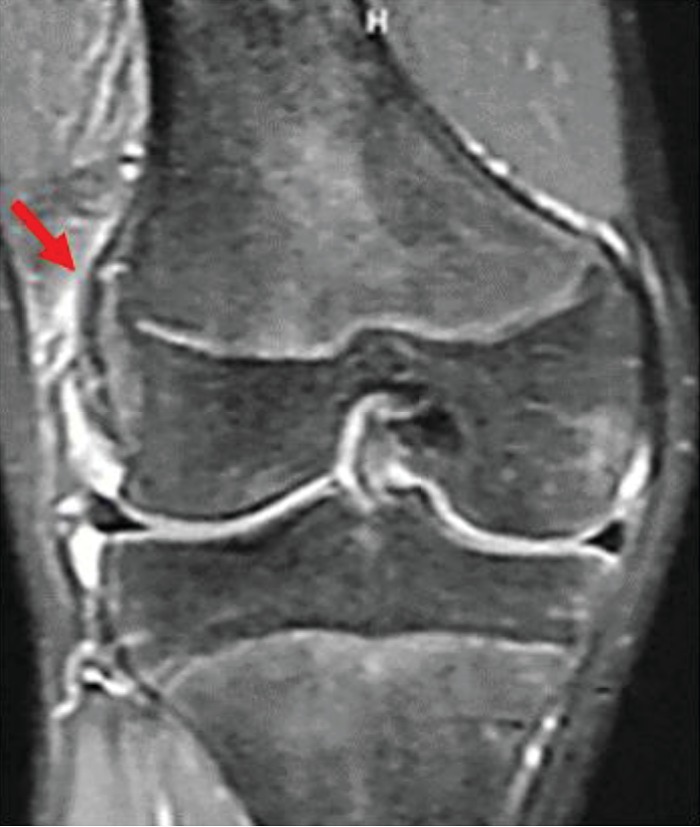
A bony bridge on the lateral side of the growth plate was noted on MRI taken at age of 16.

**Figure 7:**
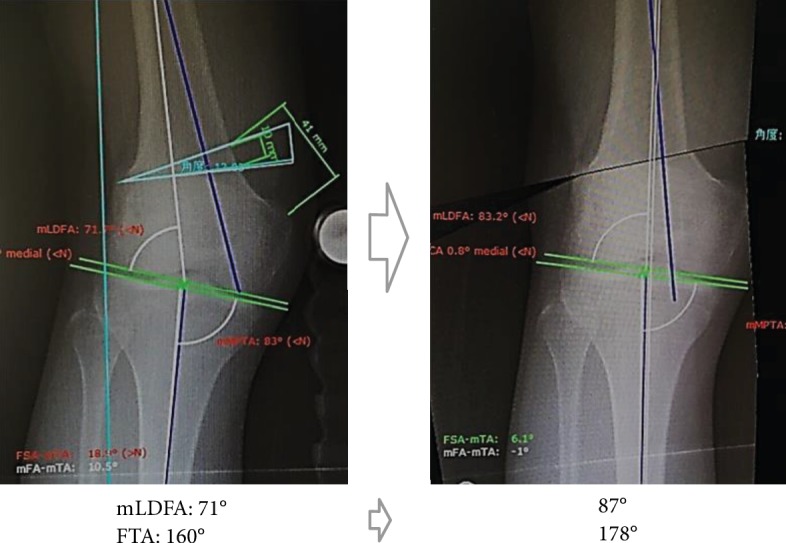
Preoperative planning. mLDFA was planned to improve to 87 degrees and FTA to 178 degrees with a correction of 16 degrees.

An 18-year-old male suffered a valgus injury to the right knee due to a fall during a bigfoot race he took part in when he was 15 years old. He visited a different hospital because of right knee pain and effusion. However, no obvious ligament injury or fracture was noted on MRI and physical examination, and the patient was followed up at the hospital. At the age of 17, the patient gradually became aware of the valgus deformity of the right knee. At the age of 18, the valgus deformity had further progressed, and the patient visited our department due to continuous right knee pain. There were no comorbidities in particular and no past medical history of note. At the time of his initial visit, the patient was the manager of a high school baseball club. A physical examination revealed that the valgus deformity of the right knee was visibly obvious (Figure 1). Joint effusion of the right knee and tenderness on the lateral condyle of the femur were noted. The range of motion was 0 (extension) to 140 (flexion) degrees denoting a slight flexion restriction. Lower limb length discrepancy (spinomalleolus distance (SMD)) was −3.0 cm in the right lower limb. X-ray images at the time of the initial visit to our department showed severe valgus deformity with a femoro-tibial angle (FTA) of 160 degrees and % mechanical axis (%MA) of 100% from the medial edge of the tibial plateau (Figure 2). Valgus deformity of the right knee was pronounced with a mechanical lateral distal femoral angle (mLDFA) of 71 degrees in contrast to that of left mLDFA which was 87 degrees, whereas the medial proximal tibial angle (MPTA) was normal at 85 degrees (left: 85 degrees), suggesting that the femur side was at the center of valgus deformity. X-ray images taken at the time of injury (at age 15) showed no obvious fractures or growth plate fractures (Figure 3). MRI at the time of injury (T2-enhanced) showed mild bone marrow edema in the lateral condyle of the femur and a high signal area suggestive of hemorrhage under the muscular layer (Figure 4). X-ray images taken during the four years after the injury showed that the valgus deformity was gradually progressing each year (Figure 5). A bony bridge on the lateral side of the growth plate was noted on MRI taken at the age of 16 (Figure 6). Similar to the X-ray images, MRI performed at the age of 17 also showed a bony bridge on the lateral side of the growth plate. Based on these findings, the patient was diagnosed with a valgus deformity of the knee due to traumatic premature closure of the femoral growth plate. We decided to perform a biplane-cut medial closing wedge distal femoral varus osteotomy (DFO) using a locking plate and the central area of the deformity was the distal femur [4]. In preoperative planning, we planned to improve the mLDFA to 87 degrees and FTA to 178 degrees with a 16-degree correction (10 mm closing wedge) (Figure 7).

During preoperative arthroscopy, although the anterior cruciate ligament and the lateral compartment were normal, International Cartilage Research Society (ICRS) grade II to III cartilage damage was noted in the patellofemoral joint. The minimally invasive plate osteosynthesis (MIPO) technique was used for DFO. A 4–5 cm longitudinal incision was made at the lateral side of the femur just above the femoral epicondyle according to the biplanar technique. The TomoFix medial distal femur (MDF) anatomical plate (DePuy Synthes, Solothurn, Switzerland) was bent according to the individual's anatomy and positioned under the vastus medialis muscle for osteotomy fixation [5]. Postoperative X-ray images showed that the operation went almost as planned with an improvement of 86 degrees in mLDFA and 177 degrees in FTA. Regarding postoperative therapy, range of motion exercise was started one day after surgery, partial weight-bearing exercise was permitted three weeks postoperatively and full weight-bearing exercise four/five weeks postoperatively, and the patient started jogging four months postoperatively.

**Figure 8:**
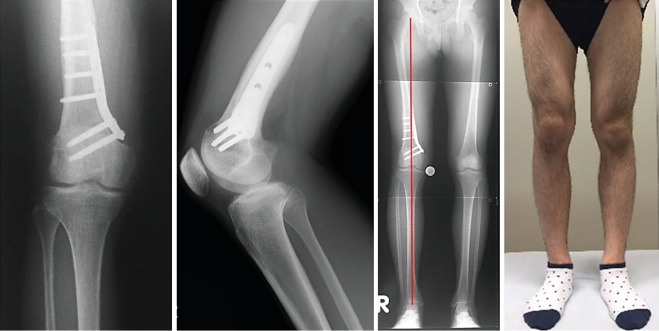
X-ray images and appearance of the lower limb six months after surgery. Bone fusion is achieved and %MA is 48.5% from the medial edge of the tibial plateau. Valgus deformity has improved in appearance.

At six months follow-up, bony fusion was achived and %MA was 48.5% from the medial edge of the tibial plateau. Bony fusion is achieved and %MA is 48.5% from the medial edge of the tibial plateau. Valgus deformity has improved in appearance, whereas lower limb length discrepancy (SMD) improved to -0.5 cm in the left lower limb and the range of motion of the right knee also improved to 0 (extension) to 150 (flexion) degrees (Figure 8). At the latest follow-up visit at 2 years after surgery, with respect to clinical scores, both the Knee injury and Osteoarthritis Outcome Score (KOOS) and International Knee Documentation Committee (IKDC) subjective score improved from 318 (before surgery) to 447 and 52 (before surgery) to 86, respectively. The KOOS showed that there was a significant improvement in symptoms, pain, sports, and quality of life.

## 3. Discussion

The incidence of femoral distal growth plate fractures is considered to be approximately 1 to 6% of all growth plate fractures [6]. Among them, Salter-Harris (SH) type II is the most common, making up about half of growth plate fractures, whereas types IV, V, and VI (Rang's type VI) are rare, accounting for only a few percent [3, 7]. Havranek and Pesl [3] reported patients treated for Rang's type VI physeal injury constituted 36 children (0.12%) of 29878 children with acute fractures with mean age was 11.6 years. In the present case, we believe SH (Rang) type VI perichondral ring injury was initially present because the fracture was caused by a valgus injury and hemorrhage around the peripheral structure of the growth plate and no obvious signs of a fracture were noted on MRI. Many previous authors have attempted to quantify risk factors for the possibility of a growth arrest occurring, including sex, SH types (II, V, and VI), age, displacement, treatment, and ligamentous laxity [3, 6, 8]. Premature closure of the growth plate is recognized by the appearance of a bony bridge in the fractured growth plate. The bony bridge is considered the key in diagnosis [3, 7, 9]. With respect to treatment, although elongation surgery of the affected limb is chosen for growing patients, long-term follow-up is recommended in some cases as spontaneous correction in some cases have been reported. Corrective osteotomy is indicated once growth has stopped [10]. In the present case, although shortening of the bony length was a concern when performing DFO with a large correction angle, we placed emphasis on good bone fusion and stability and selected medial closing wedge DFO as postoperative leg length discrepancy was about -0.5 cm according to preoperative planning. Although there is a study reporting that there was no difference in bone fusion, satisfaction, or leg length between lateral opening wedge DFO and medial closing wedge DFO [10, 11], there is also a study reporting that there were more cases of lateral opening wedge DFO that required removal of the plate due to strong irritative symptoms to the iliotibial band [12].

## 4. Conclusion

Distal femoral growth plate fractures are not benign fractures and should not be treated as such. Patients and their families should be educated and warned about the possibilities of complications, which are not limited to growth disturbance alone. We recommend that these patients be followed for several years, ideally until skeletal maturity.

## References

[1] Graham J. M., Gross R. H. (1990). Distal femoral physeal problem fractures. *Clinical Orthopaedics and Related Research*.

[2] Eid A. M., Hafez M. A. (2002). Traumatic injuries of the distal femoral physis. Retrospective study on 151 cases. *Injury*.

[3] Havranek P., Pesl T. (2010). Salter (Rang) type 6 physeal injury. *European Journal of Pediatric Surgery*.

[4] Brinkman J.-M., Hurschler C., Staubli A. E., van Heerwaarden R. J. (2011). Axial and torsional stability of an improved single-plane and a new bi-plane osteotomy technique for supracondylar femur osteotomies. *Knee Surgery, Sports Traumatology, Arthroscopy*.

[5] Nakayama H., Iseki T., Kanto R. (2018). Physiologic knee joint alignment and orientation can be restored by the minimally invasive double level osteotomy for osteoarthritic knees with severe varus deformity. *Knee Surgery, Sports Traumatology, Arthroscopy*.

[6] Canale S. T. (2003). Fractures and dislocations in children. *Campbell’s Operative Orthopaedics*.

[7] Jaramillo D., Laor T., Zaleske D. J. (1993). Indirect trauma to the growth plate: results of MR imaging after epiphyseal and metaphyseal injury in rabbits. *Radiology*.

[8] Arkader A., Warner W. C., Horn B. D., Shaw R. N., Wells L. (2007). Predicting the outcome of physeal fractures of the distal femur. *Journal of Pediatric Orthopedics*.

[9] Beck A., Kundel K., Rüter A. (1997). Significance of corrective growth of opposite physes in the surgical correction of deformity following epiphyseal injury around the knee joint. *Knee Surgery, Sports Traumatology, Arthroscopy*.

[10] Madelaine A., Lording T., Villa V., Lustig S., Servien E., Neyret P. (2016). The effect of lateral opening wedge distal femoral osteotomy on leg length. *Knee Surgery, Sports Traumatology, Arthroscopy*.

[11] Wylie J. D., Jones D. L., Hartley M. K. (2016). Distal femoral osteotomy for the valgus knee: medial closing wedge versus lateral opening wedge: a systematic review. *Arthroscopy*.

[12] Jacobi M., Wahl P., Bouaicha S., Jakob R. P., Gautier E. (2011). Distal femoral varus osteotomy: problems associated with the lateral open-wedge technique. *Archives of Orthopaedic and Trauma Surgery*.

